# Changes of DNA Methylation Pattern in Metabolic Pathways Induced by High-Carbohydrate Diet Contribute to Hyperglycemia and Fat Deposition in Grass Carp (*Ctenopharyngodon idellus*)

**DOI:** 10.3389/fendo.2020.00398

**Published:** 2020-07-10

**Authors:** Wen-Jing Cai, Xu-Fang Liang, Xiao-Chen Yuan, Ai-Xuan Li, Shan He

**Affiliations:** ^1^Chinese Perch Research Center, College of Fisheries, Huazhong Agricultural University, Wuhan, China; ^2^Innovation Base for Chinese Perch Breeding, Key Lab of Freshwater Animal Breeding, Ministry of Agriculture, Wuhan, China

**Keywords:** whole-genome DNA methylation analysis, metabolism, high carbohydrate intake, grass carp, hyperglycemia

## Abstract

Although studies have determined that epigenetics plays an essential role in regulating metabolism in mammals, research on nutrition-related DNA methylation remains to be lacking in teleosts. In the present study, we provided a hepatic whole-genome DNA methylation analysis in grass carp fed with moderate- or excessive-carbohydrate-level diet. Although a high-carbohydrate (HC) diet significantly changed the mRNA expression levels of metabolic genes, it did not affect the global genomic DNA methylation levels in grass carp liver. However, compared with the control group, 3,972 genes were hyper-methylated and 2,904 genes were hypo-methylated in the promoter region. Meanwhile, 10,711 genes were hyper-methylated and 6,764 genes were hypo-methylated in the gene body region in the HC group. These differentially methylated genes (DMGs) were enriched in multiple pathways, including carbohydrate metabolism, insulin pathway, lipid metabolism, and adipocytokine signaling pathway. In addition, the variations in DNA methylation significantly regulated the transcription levels of key genes of metabolism, which could affect the glucose concentrations and the lipid deposition of grass carp. Furthermore, we compared the DNA methylation alterations of genes in glucose metabolism and obesity pathways of grass carp with those of mammalian models in different nutritional states. The results showed that most of the DMGs in grass carp were also regulated by DNA methylation in mammals when the nutritional state changed. The findings revealed more differentially methylated regions and candidate genes for glucose metabolism and broken species boundaries.

## Introduction

Carbohydrate acts as the primary substance of metabolism in vertebrates, especially in mammals. However, many fish are considered as glucose intolerant, especially carnivorous fish. After having been fed with excessive carbohydrate, they exhibit a persistent postprandial hyperglycemia, appetite decrease, and subsequently a decrease in growth rate and even a “fatty liver.” Carnivorous fish, such as rainbow trout (*Oncorhynchus mykiss*), cannot tolerate more than 20% of digestible carbohydrate in their diet (1). However, intolerance to glucose is not apparent in herbivorous and omnivorous fish. They possess greater ability to digest and utilize dietary carbohydrate compared to carnivorous fish. Despite all that, carbohydrate is usually regarded as an economical food source because of its protein-sparing effect on reducing dietary protein contents in aquafeed (2).

In recent years, remarkable progress has been made in building fish species as diabetes research models (3). A diet-induced obese zebrafish model exhibited symptoms of type 2 diabetes (T2D) such as hyperinsulinemia and impaired glucose tolerance (4). Other non-traditional fish species have also been used as models for diabetes studies. Tilapia (*Oreochromis niloticus*) and goby (*Gillichthys mirabilis*) have been established as type 1 diabetes models (5, 6). Blind cavefish (*Astyanax mexicanus*) is born with insulin-resistant and diabetes-like phenotypes without the usual complications. A study on blind cavefish provided potential insights into better controlling high blood glucose in diabetes patients (7). Glucose/carbohydrate metabolism and utilization continues to be a contentious issue in fish as an economical food source and diabetes research models.

Recently, attention has been paid to exploring the function of epigenetics in the glucose metabolism and obesity, especially DNA methylation. Obese rats fed with high-fat diet revealed a hypermethylation of hepatic glucokinase (*gck*) and pyruvate kinase (*pk*) genes in the promoter region, suggesting the relevance between metabolic syndrome and epigenetic regulation (8). High fructose intake induced a hypermethylation of perixisome proliferation-activated receptor alpha (*ppar*α) and carnitine palmitoyltransferase 1a (*cpt1a*) genes in the promoter region in rat liver (9). DNA methylation influenced phenotype transmission and development of diseases, including T2D. In the pancreas of T2D patients, hypermethylation of peroxisome proliferator-activated receptor gamma coactivator 1 alpha (*pgc1a*), pancreatic pdx l (*pdx1*), and insulin (*ins*) genes decreased the mRNA expression levels of these genes (10–12). Volkov described the comprehensive features of DNA methylome in the pancreatic islet of humans with or without diabetes (13). The studies discovered novel diabetes-related changes of DNA methylation and suggested the importance of epigenetics in T2D. Ma and Kang demonstrated the relationship between environmental factors and DNA methylation regulation, concluding that differential methylated regions and genes are associated with obesity and T2D (14). Although studies have determined metabolism-associated epigenetic variations in mammals, research on nutrition-related DNA methylation remains to be lacking in teleosts.

The production of grass carp (*Ctenopharyngodon idella*) composes the largest aquaculture industry in China. As a typical herbivorous fish, it possesses greater capability to utilize carbohydrate compared with omnivorous and carnivorous fish (15). Gao et al. (16) revealed that the growth rates of grass carp were maximal when the dietary carbohydrate level was 274.7 g kg^−1^. According to our previous studies, the concentrations of serum glucose and insulin were significantly increased in grass carp when the dietary carbohydrate level was 42% (17). A high-carbohydrate diet (more than 40%) also increased serum cholesterol and total lipid contents; however, it suppressed appetite in grass carp (18). To address the knowledge gap of glucose metabolism and obesity-related epigenetics in fish, our study provided an in-depth analysis of whole-genome DNA methylation of grass carp liver. Furthermore, we compared the DNA methylation alterations of key genes related to glucose metabolism with those of other animal models to reveal novel differentially methylated regions and candidate genes for more information on disorders of glucose metabolism.

## Materials and Methods

### Experimental Diets

The formulation of diet was designed based on our previous research. Different diets which satisfied the nutritional requirement of grass carp but with varied carbohydrate levels (25.00 and 42.00 g 100g^−1^, named as control group and HC group) were produced (17). Table 1 displays the composition and the chemical component analysis of the two diets. All the ingredients used in the diet were purchased from Fulong Dietary Company (Wuhan, China). The ingredients were finely ground, fully mixed, and dry-pelleted into 1.5-mm pellets using a pellet mill.

**Table 1:** Composition of the experimental diets.

**Item**	**Control**	**High carbohydrate**
**Ingredient (%)**		
Casein	32	32
Gelatin	8	8
Fish oil	3	3
Soybean oil	3	3
Corn starch	25	42
Cellulose	22.8	5.8
Vitamin mixture^a^	1	1
Mineral mixture^b^	1	1
Ca(H_2_PO_4_)_2_	1.65	1.65
Carboxymethyl cellulose	2	2
Choline chloride (50%)	0.5	0.5
Ethoxyquin (30%)	0.05	0.05
**Compositions (%)**		
Crude protein	36.96	37.14
Crude lipid	6.35	6.24
Carbohydrate	25.21	45.30
Ash	2.50	2.84
Cross energy (kJ g^−1^)	11.87	15.12

a *Vitamin premix (per kilogram of diet): vitamin A, 2,000 IU; vitamin B1 (thiamin), 5 mg; vitamin B2 (riboflavin), 5 mg; vitamin B6, 5 mg; vitamin B12, 0.025 mg; vitamin D3, 1,200 IU; vitamin E, 21 mg; vitamin K3, 2.5 mg; folic acid, 1.3 mg; biotin, 0.05 mg; pantothenic acid calcium, 20 mg; inositol, 60 mg; ascorbic acid (35%), 110 mg; and niacinamide, 25 mg*.

b *Mineral premixes (per kilogram of diet): MnSO_4_, 10 mg; MgSO_4_, 10 mg; KCl, 95 mg; NaCl, 165 mg; ZnSO_4_, 20 mg; KI, 1 mg; CuSO_4_, 12.5 mg; FeSO_4_, 105 mg; Na_2_SeO_3_, 0.1 mg; and Co, 1.5 mg*.

### Experimental Conditions and Sample Collection

Experimental grass carp were purchased from the Fish Center in Xiantao, Hubei Province, China, and kept in 1,000-L tanks with constant flow of filtered water. The fish were fed twice a day at 9:00 a.m. and 6:00 p.m. for 2 weeks to be acclimated to the feeding conditions. After acclimation, the fish (196.82 ± 2.64 g) were randomly distributed into six 1,000-L tanks (three tanks per group) with 15 fish per cage. During the experiment, the fish were fed with equal quantities of the experimental diets at 9:00 a.m. and 6:00 p.m. for 6 weeks. At 2 h after feeding, the residual diets were collected and dried at 60°C and then weighed for calculation of food intake. At the end of the feeding trial, all the fish were anesthetized with MS-222 and weighed ~24 h after the last feeding. In each group, six fish were randomly captured and dried at 60°C or frozen at −20°C until the analysis of body composition. Another six fish were randomly captured; the small pieces of liver were immediately collected and frozen in liquid nitrogen and stored at −80°C for isolation of DNA and RNA. Blood was collected from the caudal vein and centrifuged at 3,500 g for 30 min, and then the serum was separated and stored at −80°C until use.

The crude lipid was determined based on ether extraction method by Soxtec System HT (Soxtec System HT6, Tecator, Sweden). The glucose contents were determined using an automatic biochemical analyzer (Abbott Aeroset Analyzer, USA) in Zhongnan Hospital of Wuhan University (Wuhan, Hubei, China). The insulin levels were detected by enzyme linked immunosorbent assay (ELISA) based on the manual of the Fish Insulin ELISA kit (Xinle, Shanghai, China).

### Extraction of DNA and Bisulfite Conversion

The hepatic genomic DNA of three grass carp in each group was extracted using TIANamp Genomic DNA Kit (Tiangen, Beijing, China) in accordance with the standard procedures. An Eppendorf BioPhotometer (Hamburg, Germany) and 1.5% agarose gel electrophoresis were used to detect the integrity and the quantity of genomic DNA.

### Library Preparation and Quantification

For whole-genome bisulfite sequencing (WGBS) library construction, the genomic DNA of grass carp liver was fragmented by sonication with a Bioruptor (Diagenode, Belgium) to an average size of around 250 bp, followed by having it end-repaired, 3-end-adenylated, and ligated with adapters, essentially based on the instructions of the manufacturer. Using EZ DNA Methylation-Gold kit (ZYMO), the ligated DNA was bisulfite-converted. Fragments were extracted from the same lane of 2% agarose gel. Lastly, the products were purified with QIAquick Gel Extraction kit (Qiagen) and amplified by PCR.

### The Whole-Genome Bisulfite Sequencing and Identification of DMRs

The libraries were sequenced on Illumina Hiseq Xten platform. The peak signal was transformed into base sequence by base calling as raw reads. The data of raw reads were filtered to guarantee the quality, including the removal of reads with more than 10% N content or more than 10% low-quality bases. The final filtered data were called clean reads.

### Mapping Reads to the Grass Carp Genome Database

After filtering, the clean reads were mapped to the reference genome (http://www.ncgr.ac.cn/grasscarp/) by the Bisulfite Sequence Mapping Program. Then, the duplication reads were removed, and the mapping rate and the bisulfite conversion rate of each sample were calculated.

### Identification of Methylation Levels and Differentially Methylated Regions

DNA methylation level was calculated by dividing the read number of each methyl-cytosine (mC) by the total read number of cytosines, equal to the mC/C ratio at each reference cytosine. The methylation levels of the C site were counted as Nm/(Nm + Nnm). Nm represented the reads of mC, while Nnm meant the non-methylation reads.

The differentially methylated regions (DMRs) between two groups of grass carp were identified by comparison of the sample methylomes through windows which are composed of five CpG sites, at least with a twofold change in the methylation level and with *p* ≤ 0.05 (Fisher test). In addition, two neighboring DMRs were joined into one continuous DMR if the genomic methylation level of the region from the start of upstream DMR to the end of downstream DMR also showed twofold differences between the two groups. Otherwise, the two DMRs were considered as independent.

### GO and KEGG Enrichment Analysis of Genes Related to DMRs

Genes that overlapped with DMRs were termed as differentially methylated genes (DMGs). DNA methylation in different gene sub-elements plays different roles in gene expressions. Thus, in our research, Gene Ontology (GO) and Kyoto Encyclopedia of Genes and Genomes (KEGG) pathway analyses were separated in DMGs whose DMR was located in the promoter region or in the gene body. The GO analysis of DMGs was as follows: Firstly, we mapped all DMRs to GO terms in the database (http://www.geneontology.org/), calculating the gene numbers for every term. Then, we searched for significantly enriched GO terms in the input list of DMRs based on GO TermFinder (http://www.yeastgenome.org/help/analyze/go-term-finder). The method used can be described as follows:

$$\begin{array}{l} \text{p}=1-\overset{m-1}{\underset{i=0}{\sum }}\frac{\left(\begin{array}{c} M\\ N \end{array}\right)\left(\begin{array}{c} N-M\\ n-i \end{array}\right)}{\left(\begin{array}{c} N\\ n \end{array}\right)} \end{array}$$

where *N* is the number of all genes with GO annotation, *n* is the number of DMRs in *N, M* is the number of all genes that are annotated to certain GO terms, and *m* is the number of DMRs in *M*. The calculated *p*-value goes through Bonferroni correction. GO terms with corrected *p* ≤ 0.05 were considered as significantly enriched.

The statistical enrichment of DMGs in KEGG pathways was conducted with KOBAS software (19). The pathways with corrected *p* ≤ 0.05 were determined as significantly enriched.

### RNA Isolation and Reverse Transcription

The hepatic total RNA of six fish in each group was extracted using Trizol Reagent (TaKaRa, Japan). The quantity and the integrity of RNA were determined by an Eppendorf BioPhotometer and agarose gel electrophoresis method (Hamburg, Germany). The hepatic cDNAs of grass carp were achieved from 1 μg total RNA according to the instructions of HiScript® II Reverse Transcriptase (Vazyme Biotech, China).

### Quantification of the Hepatic Transcript of Differentially Methylated Genes

The genes of key enzymes that participated in glucose and fatty acid metabolism, the transcription factors of the above-mentioned genes, and the critical genes in insulin/leptin/AMPK pathways were selected as the candidate genes for an analysis of mRNA levels. If the gene was differently methylated in the promoter region or in the gene body region, the mRNA expression levels would be detected on a quantitative thermal cycler (CFX96 Real-Time PCR Detection System, Bio-Rad, USA). Information on the primers used in this experiment is shown in Table 2. The PCR cycling parameters were 95°C for 3 min, followed by 40 cycles at 95°C for 10 and 30 s at annealing temperature. After amplification, melting curve assays were performed. The temperature gradually increased from 65 to 95°C, with 0.5°C every 6 s. According to the instructions of GoTaq® qPCR Master Mix (Bio-Rad, USA), the reaction was composed of 0.2 μM of primers, 1 μl cDNA, 10 and μl GoTaq® qPCR Master Mix and supplied with double-distilled water at 20 μl. The mRNA expression levels were quantified relative to the expression of elongation factor 1 α (*ef1*-α) as endogenous reference (20) and analyzed by the optimized comparative Ct (2^ΔΔ*Ct*^) value method (21).

**Table 2:** Primer sequences for the quantitative real-time PCR.

**Gene**	**Primers**	**Sequence 5′-3′**	**Accession number**
pepck	pepck-F	ATCGTCACGGAGAACCAA	JQ898294
	pepck-R	CCTGAACACCAAACTTAGCA	
gs	gs-F	CCTCCAGTAACAACTCACAACA	JQ792167
	gs-R	CAGATAGATTGGTGGTTACGC	
pk	pk-F	GCCGAGAAAGTCTTCATCGCACAG	KP262353.1
	pk-R	CGTCCAGAACCGCATTAGCCAC	
g6pca.1	g6pca.1-F	CTACAACGCAAGTCTGAGAAAGT	MN176655
	g6pca.1-R	CAGTCTGGATTGACGCACC	
acc1	acc1-F	TGGCTGCACTGCACTCTCACT	GU908475
	acc1-R	GGTCCAGCTTCCCTGCGGTC	
fasn	fasn-F	GATGGGTCTACAGCCTGATGG	HM802556
	fasn-R	GACACCCTGTGGACATTGAGC	
cpt1	cpt1-F	GCCACTGTAAAGGAGAACC	JF728839
	cpt1-R	GGATGCCTCATAAGTCAAG	
atgl	atgl-F	TCGTGCAAGCGTGTATATG	HQ845211
	atgl-R	GCTCGTACTGAGGCAAATTA	
pparα	pparα-F	AGCAGAGAAGGACGTCAG	FJ595500
	pparα-R	TTCCTTCTCGGCATGCTG	
srebp1	srebp1-F	CTTCCTCTTGTTCGCCTGCT	KJ162572.1
	srebp1-R	CCTTTTGCCATACCTCTGCC	
pgc α	pgc1α-F	CAGGAAACTACTAAGGGACCAG	HM015283
	pgc1α-R	TGAGATGAGGAACGGGAGC	
insrb	insrb-F	GTCCACCACCAACCCTGAA	KR866114
	insrb-R	TCCCGCCCTTGCGATAAT	
foxo1	foxo1-F	GCTTGAACTGGTGTCGGTCTC	KP325483.1
	foxo1-R	GCCGCTCGTCCTCTGCTC	
socs3	socs3-F	GCTGCCGTCTCACCGTTAC	MN176655
	socs3-R	ATGCTCTTGGAGTCCGTTTGT	
ampkα	ampkα-F	GTCCAAGCATCTCGGTGTTC	MK294331.1
	ampkα-R	GGGTTCTTCCTCCGCACT	
leptin a	leptin a-F	TGAGCATTCTTGGTATGATTGA	FJ373293
	leptin a-R	ATTCTGTGGATGATGGTGTCTG	
ef1α	ef1α-F	GCTGACTGTGCCGTGCTGAT	GQ266394
	ef1α-R	GCTGACTTCCTTGGTGATTTCC	

### *In vitro* Analysis of the Effects of 5-Aza-2′-deoxycytidine on Glucose Consumption

Grass carp liver cell line (L8824) was obtained from Wuhan Cell Instruction Repository (Wuhan, Hubei, China). The cells were seeded in 24-well culture plates, at a density of 10^5^ cells/well, and cultured in Medium 199 (Cat. No. C11150500BT, gibco) supplemented with 10% fetal bovine serum (Sigma-Aldrich, Saint Louis, MO) at 28°C in 5% CO_2_. After 24 h, the cells were treated with different concentrations of glucose (5, 15, and 30 mM) and a DNA methyltransferase inhibitor (0 and 10 μM), 5-Aza-2′-deoxycytidine (Product Number A3656, Sigma-Aldrich), for 48 h. For each treatment, three biological replicates were set. The glucose concentration of the cell culture medium was detected at 0 and 48 h after treatment by a glucose assay kit (glucose oxidase method; ROBIO, Shanghai). The glucose concentration difference between 0 and 48 h was considered as the glucose consumption level of L8824 cells.

### Statistical Analysis

Data on growth performance, food intake, serum components, target DMG expression levels, and *in vitro* analysis of the effects of 5-Aza-2′-deoxycytidine on glucose consumption were analyzed with SPSS 19.0 software (SPSS, USA). The differences were assessed using independent-samples *t*-test. Statistical significance was considered at *p* < 0.05. All data were presented as mean ± S.E.M (standard error of the mean). GraphPad Prism 8 software was employed to generate the figures. The whole-genome bisulfite sequencing library, identification of DMRs, GO annotation, and KEGG pathway enrichment of DMGs were operated by BGI Genomics Co., Ltd. (Shenzhen, China). The figures of DMG enriched pathways involved in metabolism were generated with ScienceSlides and Powerpoint 2010.

## Results

### Growth Performance and Serum Components

**Figure 1:**
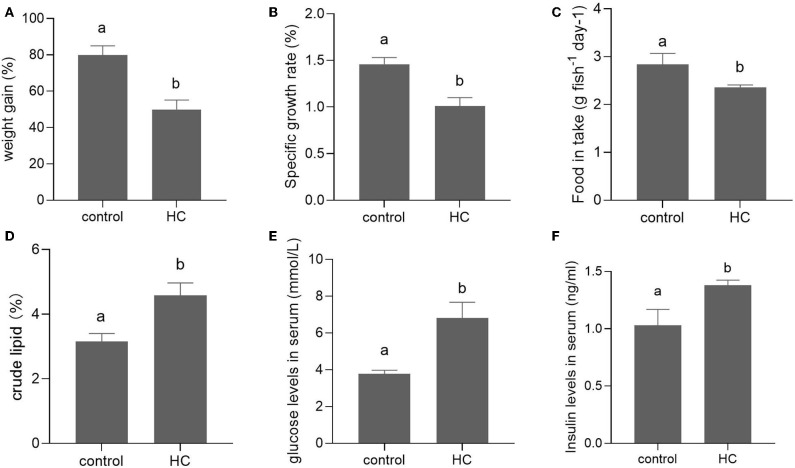
Growth performance and serum components in grass carp fed with moderate carbohydrate diet (control) or excessive carbohydrate diet. **(A)** Weight gain. **(B)** Specific growth ratio. **(C)** Food intake. **(D)** Body crude lipid contents. **(E)** Concentration of serum glucose. **(F)** Concentration of serum insulin. Values are mean ± SEM (*n* = 6). The vertical bars which are not sharing the same letter are significantly different (*P* < 0.05).

After a 6-week feeding trial, the weight gain, specific growth ratio (SGR), and food intake were significantly decreased in grass carp fed with high-carbohydrate diets (*p* < 0.05) compared with the control group. The body crude lipid contents, serum glucose, and insulin concentrations of grass carp fed with high-carbohydrate diets were significantly increased compared with the control group (*p* < 0.05; Figure 1).

### Bisulfite Sequencing and DNA Methylation Profile

In the present study, hepatic DNA methylation of grass carp fed with control and high-carbohydrate (HC) diets were sequenced using WGBS technology. An average of 36.470 Gb clean bases was generated. The sequencing data for each sample is presented in Table S1. The mapping reads and the bisulfite conversion rate of each sample are shown in Table S2. High-quality DNA methylation pictures of the two groups were achieved, and the unique mapping rates ranged from 76.42 to 83.37%. The bisulfite conversion rate of genomic DNA ranged from 99.35 to 99.61%. Strict quality control (QC) was performed for each sample on several QC terms, as illustrated in Table S3. The sequencing data of all the samples were qualified (Table S3). The clean data have been submitted to the SRA database (SRA accession: PRJNA558443).

### Genome-Wide Methylation Patterns in Grass Carp Liver

Methylation in grass carp existed in different sequence contexts, including CG, CHG, and CHH (H is A, C, or T). There were about 9.45 and 9.38% of all genomic cytosines methylated in the control and the HC groups, respectively. Meanwhile, the average level of methylated cytosines in each context was also calculated. In the control group, 81.02% of CG, 0.66% of CHG, and 0.69% of CHH were methylated. Correspondingly, 80.78, 0.77, and 0.80% of cytosines were methylated in CG, CHG, and CHH contexts in the HC group (Figure S1A). The proportion of different types of methyl-cytosine varies between species and even between different tissues of the same species, and there are specific methylation profiles under physiological changes. The proportion of methylated cytosines, including mCG, mCHG, and mCHH, is presented in Figure S1B. Whole genomic DNA was defined by seven different regions, including upstream, first exon, first intron, internal exon, internal intron, last exon, and downstream. About 80% of CG was methylated in all regions, with a slight drop near the transcription start sites (TSS). The CHG and CHH methylation patterns were with higher methylation levels at the 5′ and the 3′ flanking regions compared with the other regions (Figure S1C). In general, there were no significant differences in the CG, CHG, or CHH methylation patterns between the two groups.

### Identification of Hepatic Differentially Methylated Genes in Grass Carp Fed With Diets With Different Compositions of Carbohydrate

By a genome-wide comparison of the DNA methylation sequences in grass carp, the accepted different levels of carbohydrate (102,837 DMRs in CG context, 221 DMRs in CHG context, and 7,434 DMRs in CHH context) were identified. The distribution of differentially methylated genes is shown in Figure S2. A total of 6,403, 13, and 460 DMGs in CG, CHG, and CHH contexts in the promoter region were obtained. Meanwhile, 15,007, 66, and 2,403 DMGs in CG, CHG, and CHH contexts in the gene body region were observed (Figure S2A). Compared to the control group, 3,972 genes were hyper-methylated, while 2,904 genes were hypo-methylated in the promoter region in the HC group. Meanwhile, 10,711 genes were hyper-methylated and 6,764 genes were hypo-methylated in the gene body region in grass carp fed with high-carbohydrate diet (Figure S2B).

### GO Annotation and KEGG Pathway Enrichment Analyses of DMGs

**Figure 2:**
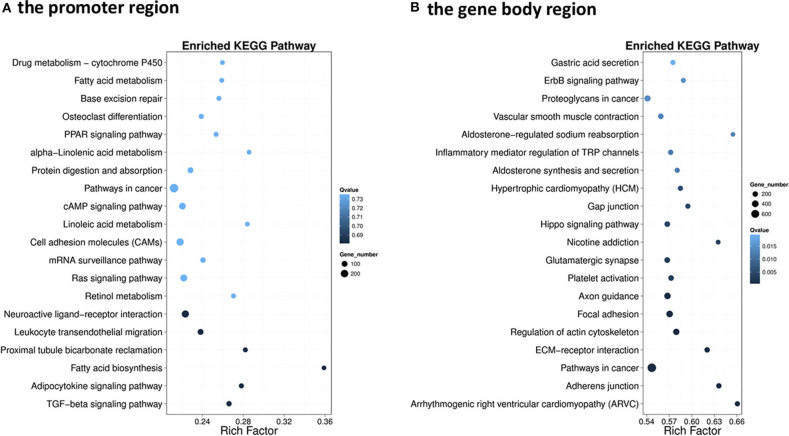
Scatterplot of the enriched top 20 Kyoto Encyclopedia of Genes and Genome pathways of differentially methylated genes (DMGs) that differentially methylated in the promoter **(A)** and the gene body regions **(B)**. The *y*-axis represents the enriched pathways, and the *x*-axis represents the rich factor of the corresponding pathways; the size of the spots represents the number of genes related to differentially methylated regions enriched in each pathway, while the color of the spot represents the corrected *p*-value of each pathway. The rich factors indicate the ratio of the number of DMGs mapped to a certain pathway to the total number of genes mapped to this pathway. Greater rich factor means greater enrichment.

**Figure 3:**
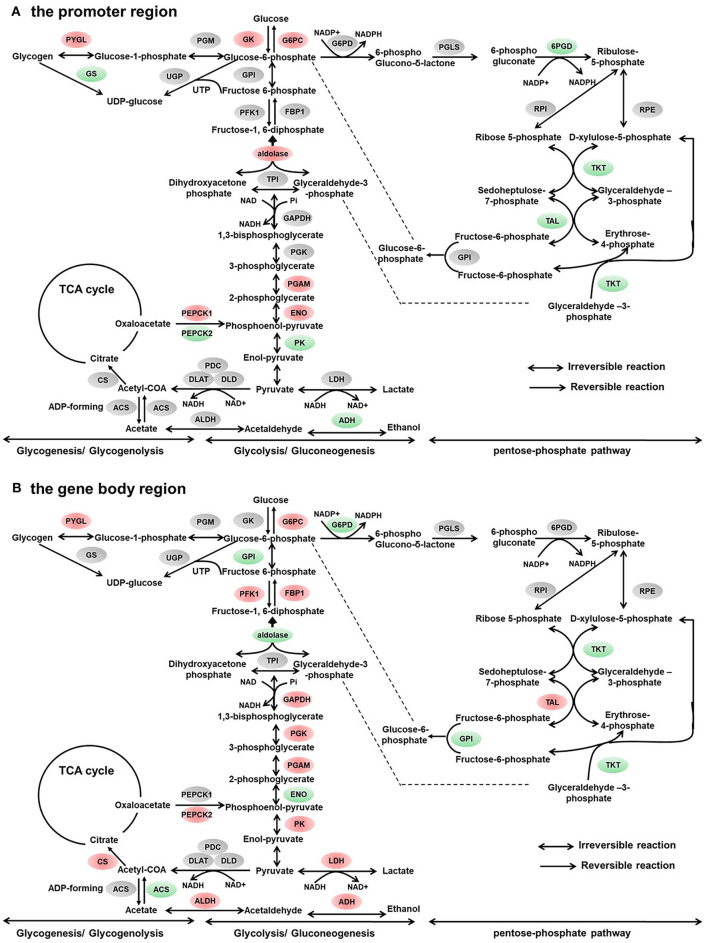
Differentially methylated genes enriched in the carbohydrate metabolism. **(A)** Genes differentially methylated in the promoter region. **(B)** Genes differentially methylated in the gene body regions. The colors of the ellipses were shaded according to significance level. Red: the DNA methylation levels of the control group were significantly higher than those in the high carbohydrate (HC) group [log2^ratio(control/HC)^ ≥ 0 and corrected *p* ≤ 0.05]. Green: the DNA methylation levels of the control group were significantly lower than those in the HC group (log2^ratio(control/HC)^ ≥ 0 and corrected *p* ≤ 0.05). Gray: not significantly changed.

**Figure 4:**
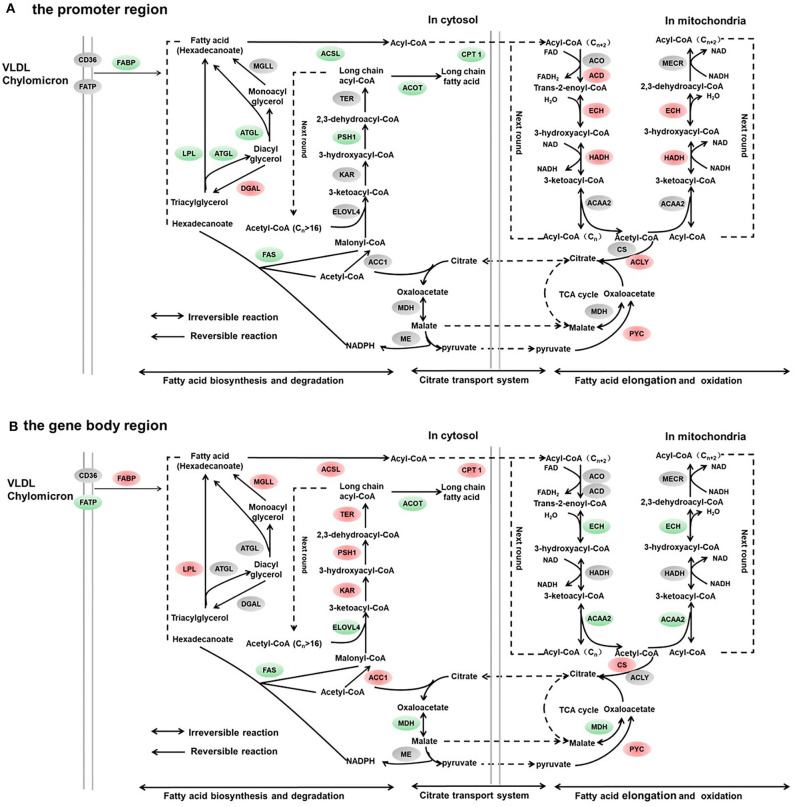
Differentially methylated genes enriched in the lipid metabolism. **(A)** Genes differentially methylated in the promoter region. **(B)** Genes differentially methylated in the gene body regions. The colors of the ellipses were shaded according to significance level. Red: the DNA methylation levels of the control group were significantly higher than those in the high carbohydrate (HC) group [log2^ratio(control/HC)^ ≥ 0 and corrected *p* ≤ 0.05]. Green: the DNA methylation levels of the control group were significantly lower than those in the HC group [log2^ratio(control/HC)^ ≥ 0 and corrected *p* ≤ 0.05]. Gray: not significantly changed.

**Figure 5:**
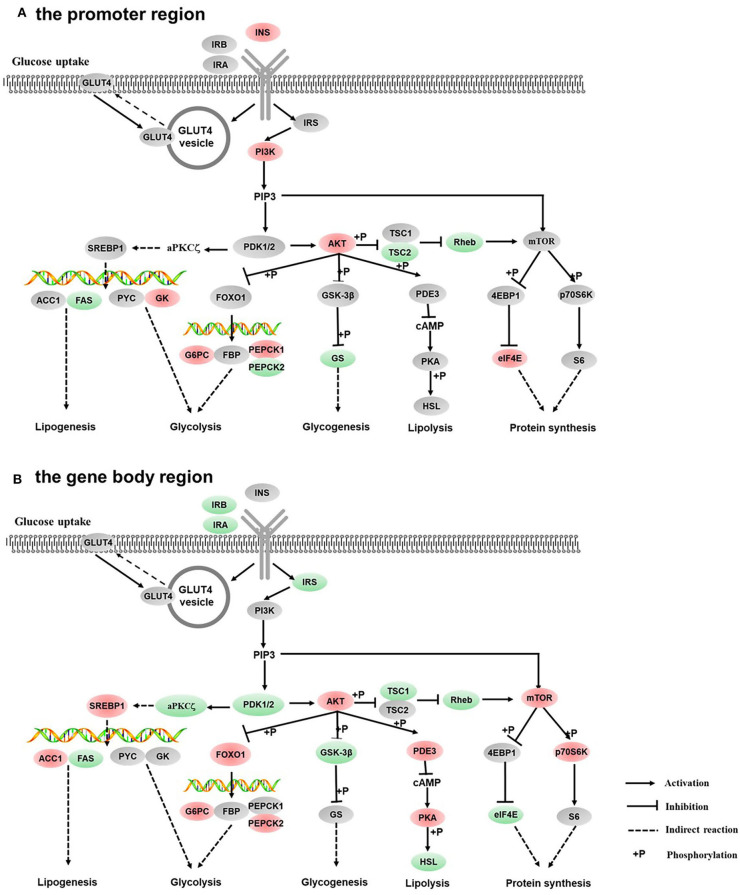
Differentially methylated genes enriched in the insulin signaling pathway. **(A)** Genes differentially methylated in the promoter region. **(B)** Genes differentially methylated in the gene body regions. The colors of the ellipses were shaded according to significance level. Red: the DNA methylation levels of the control group were significantly higher than those in the high carbohydrate (HC) group [log2^ratio(control/HC)^ ≥ 0 and corrected *p* ≤ 0.05]. Green: the DNA methylation levels of the control group were significantly lower than those in the HC group [log2^ratio(control/HC)^ ≥ 0 and corrected *p* ≤ 0.05]. Gray: not significantly changed.

**Figure 6:**
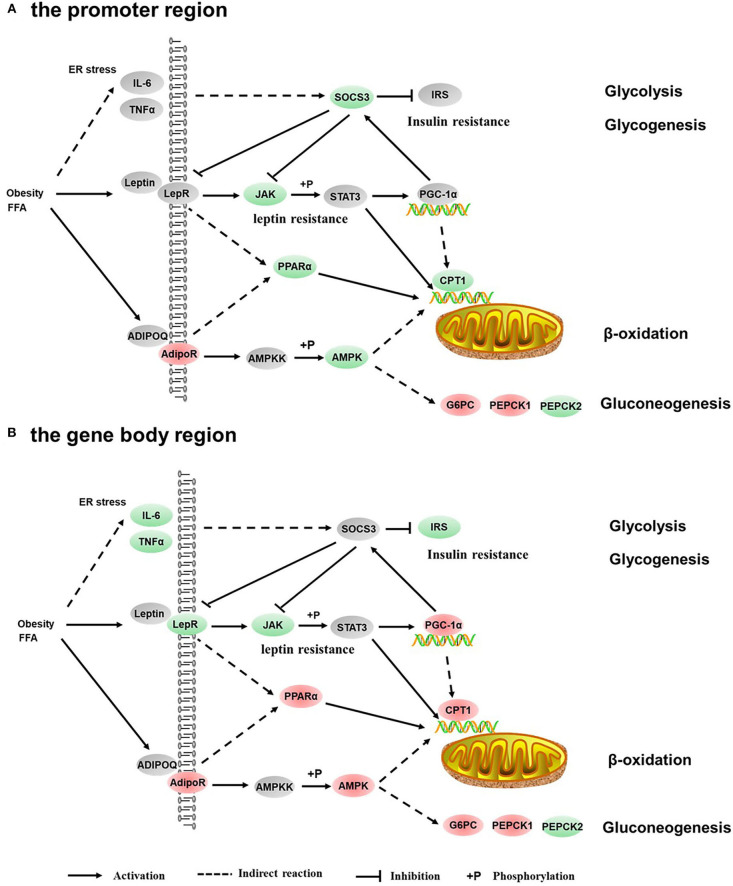
Differentially methylated genes enriched in the adipocytokine signaling pathway. **(A)** Genes differentially methylated in the promoter region. **(B)** Genes differentially methylated in the gene body regions. The colors of the ellipses were shaded according to significance level. Red: the DNA methylation levels of the control group were significantly higher than those in the high carbohydrate (HC) group [log2^ratio(control/HC)^ ≥ 0 and corrected *p* ≤ 0.05]. Green: the DNA methylation levels of the control group were significantly lower than those in the HC group [log2^ratio(control/HC)^ ≥ 0 and corrected *p* ≤ 0.05]. Gray: not significantly changed.

**Figure 7:**
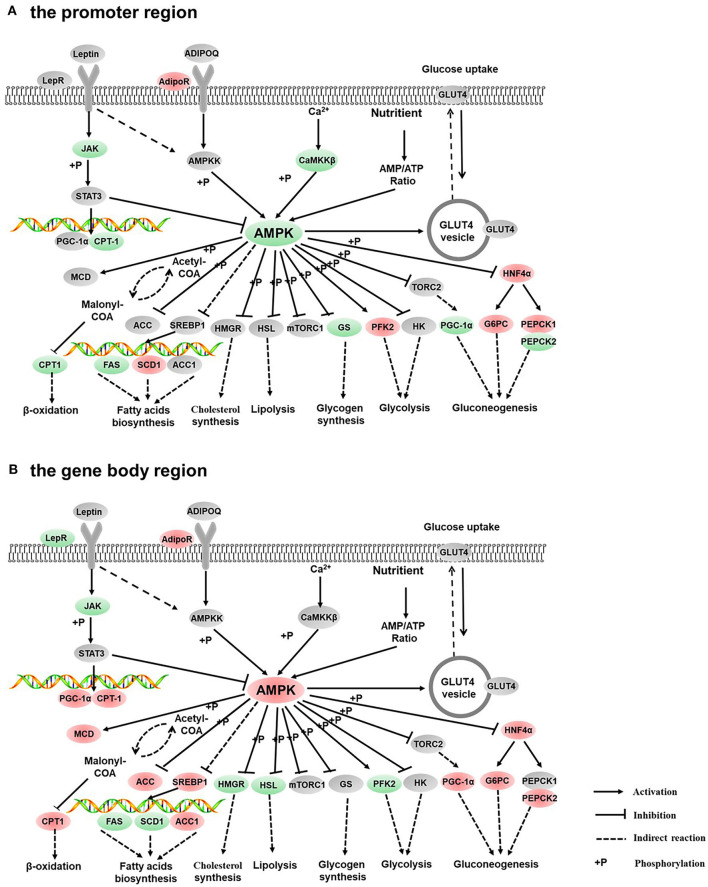
Differentially methylated genes enriched in the AMPK signaling pathway. **(A)** Genes differentially methylated in the promoter region. **(B)** Genes differentially methylated in the gene body regions. The colors of the ellipses were shaded according to significance level. Red: the DNA methylation levels of the control group were significantly higher than those in the high carbohydrate (HC) group [log2^ratio(control/HC)^ ≥ 0 and corrected *p* ≤ 0.05]. Green: the DNA methylation levels of the control group were significantly lower than those in the HC group (log2^ratio(control/HC)^ ≥ 0 and corrected *p* ≤ 0.05). Gray: not significantly changed.

The GO enrichment analysis of DMGs in CG context revealed that DMGs were significantly enriched in cellular process, biological regulation, single-organism process, metabolic process, cell part, and molecular binding (Figure S3). KEGG enrichment of DMGs was also conducted both in the promoter region and in the gene body region for CG context. In the promoter region, 302 pathways were significantly enriched (Figure S4A). The top 20 pathways in ascending order of corrected *Q*-value are displayed in Figure 2A. The results suggested that TGF-beta signaling pathway was the most enriched pathway. In addition, DMGs were significantly enriched in metabolism-related pathways, including fatty acid metabolism (ko00061), PPAR signal pathway (ko03320), protein digestion and absorption (ko04974), fatty acid biosynthesis (ko01212), and adipocytokine signaling pathway (ko04920). In the gene body region, 305 pathways were significantly enriched (Figure S4B). However, no metabolism-related pathway was enriched in the top 20 KEGG pathways (Figure 2B). Furthermore, as a continuation of a previous study (17, 18), the present study focused on the DNA methylation changes of pathways involved in the regulation of metabolism, including carbohydrate metabolism (Figure 3), lipid metabolism (Figure 4), insulin signaling pathway (Figure 5), adipocytokine signaling pathway (Figure 6), and AMPK pathway (Figure 7).

### Expression Levels of Target DMGs by Real-Time PCR

**Figure 8:**
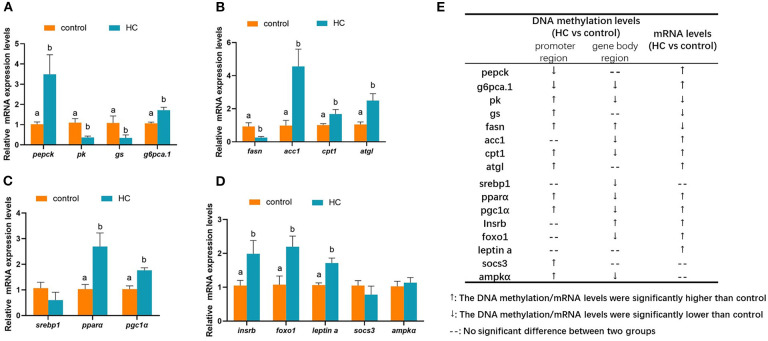
Hepatic mRNA expression levels of different methylated genes (DMGs) in grass carp fed with different carbohydrate levels. **(A)** mRNA expression levels of DMGs involved in carbohydrate metabolism. **(B)** mRNA expression levels of DMGs involved in lipid metabolism. **(C)** mRNA expression levels of different methylated transcription factor genes related to metabolism. **(D)** mRNA expression levels of DMGs in insulin pathway, adipocytokine pathway and AMPK pathway. **(E)** A summarization of the mRNA expression levels and DNA methylation pattern of the DMGs. Values are mean ± S.E.M. (*n* = 6). Vertical bars not sharing the same letter are significantly different (*p* < 0.05).

To validate the correction between DNA methylation and gene mRNA expression levels, qRT-PCR was conducted with 16 DMGs. As displayed in Figure 8A, compared with the control group, the mRNA expression levels of glycogen synthase (*gs*) and *pk* genes were significantly decreased (*p* < 0.05). However, the mRNA expression levels of phosphoenolpyruvate carboxykinase (*pepck*) and glucose-6-phosphatase a.1 (*g6pca.1*) were significantly increased (*p* < 0.05) in the HC group. The hepatic transcript levels of lipid metabolism-related genes are presented in Figure 8B. In the HC group, the hepatic transcript levels of acetyl-coA carboxylase 1 (*acc1*), *cpt1*, and adipose triglyceride lipase (*atgl*) were obviously increased when compared with the control group. However, the expression levels of fatty acid synthase (*fasn*) showed an inverse variation (*p* < 0.05). The hepatic transcript levels of the transcription factors are displayed in Figure 8C. High-carbohydrate diets significantly stimulated the mRNA expression levels of *ppar*α and *pgc1*α genes *(p* < 0.05) but did not affect the gene expression of sterol-regulatory element binding protein 1 (*srebp1*). Furthermore, the high-carbohydrate diet impacted the mRNA expression levels of signal factors in insulin signaling and adipocytokine signaling pathways (Figure 8D). The mRNA expression levels of insulin receptor b (*insrb*), forkhead box O 1 (*foxo1*), and *leptin a* were significantly increased (*p* < 0.05). The correlation between changes of the gene expression levels and the methylation levels is presented in Figure 8E.

### Effects of 5-Aza-2′-deoxycytidine on Glucose Consumption

**Figure 9:**
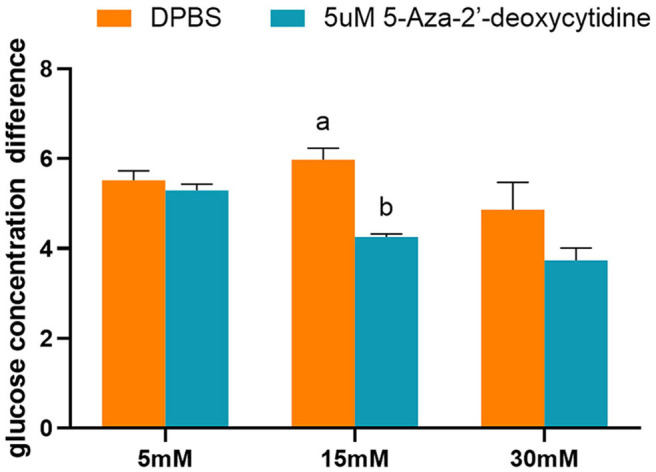
The glucose concentration difference of L8824 cell culture medium treated with different concentrations of glucose and 5-Aza-2′-deoxycytidine for 48 h. Values are mean ± SEM (*n* = 3). The vertical bars not sharing the same letter are significantly different (*p* < 0.05).

The effects of 5-Aza-2′-deoxycytidine on glucose consumption were determined by detecting the glucose concentration of the cell culture medium. The addition of 5-Aza-2′-deoxycytidine into the L8824 cell line significantly reduced the glucose consumption when the initial glucose concentration of the cell culture medium was 15 mM (*p* < 0.05). However, the glucose concentration was not affected by the DNA methylase inhibitor when the initial glucose concentration was 5 and 30 mM (Figure 9).

## Discussion

To date, most studies on epigenetics have been undertaken in mammalian species. DNA methylation is the best-known epigenetic modification. It has been proven to be sensitive to external contributors, including food nutrition in mammals (22). Different food components and dietary nutrients are associated with DNA methylation that might contribute to obesity and other metabolic disorders in mammals (23). Palmitic acid increased the global DNA methylation levels of human pancreatic islets, reducing the secretion of insulin and raising the risk of T2D as a result (24). Addition of saturated fatty acids and elaidic acid to a diet induced the hypomethylation of tumor necrosis factor and the hypermethylation of pparγ, regulating the gene mRNA expression levels and inducing inflammation in adipose tissue (25). In teleosts, although studies examining the role of epigenetics in regulating the phenotypic responses to dietary nutrition have been performed in rainbow trout and European sea bass (26, 27), the knowledge of epigenetics in fish is still in its infancy.

Grass carp is considered as a promising research model for improving the capacity of carbohydrate utilization in fish species. In the present study, by the inhibition of DNA methyltransferase in a grass carp liver cell line L8824, we confirmed that DNA methylation could mediate the glucose consumption of liver cells. To address the knowledge gap of nutrition-related epigenetics in fish, especially glucose metabolism and obesity-related epigenetics, hepatic genome-wide methylation analysis was performed in grass carp fed with suitable and excessive carbohydrates. According to our previous studies, a high-carbohydrate diet (more than 40%) intake increased the insulin concentrations and induced high serum glucose, cholesterol, and total lipid contents (17, 18). Thus, we hypothesized that a high-carbohydrate diet (more than 40%) which induced hyperglycemia and fat deposition could be compared to metabolic syndrome in humans. Integrative comparative genome-wide methylation would not only provide a comprehensive view on the epigenetic mechanisms in fish but also expand the knowledge about human T2D and related metabolic diseases.

In grass carp, the genome-wide methylation patterns were similar between two groups. Approximately 9% of all total cytosines and 80% of CG were methylated. In zebrafish, about 12% of total cytosines were methylated; 86–87% of cytosines were methylated in CG context (28). The methylation patterns of hybrid tilapias (*O. niloticus* × *Oreochromis mossambicus*) were different from those of grass carp. In skeletal muscles of hybrid tilapias, ~69.60% of cytosines in CG context were methylated. The CG methylation levels decreased gradually to 25% nearby TSS before sharply increasing to 75% in the gene body region (29). In grass carp, whole genomic DNA was defined as seven different regions, and about 80% of CG was methylated in all regions, with a slight drop near to TSS. The high level of DNA methylation in CG content was a specific characteristic of animals. DNA methylation in CHH and CHG patterns is a major characteristic of plant methylomes and largely absent in animal methylomes (30).

In juvenile rainbow trout, hypoxia exposure in the embryonic period and a high-carbohydrate diet intake at first feeding caused a long-term programming effect on the transcript of genes related to glucose metabolism, but it did not induce a significant difference in global DNA methylation in either liver or muscle tissue (31). Similar to rainbow trout, although the mRNA expression levels of glucose metabolism-related genes were significantly changed by dietary carbohydrate contents, there was no significant difference in the global genomic DNA methylation levels in grass carp liver between the two groups. Despite that, the numbers of differentially methylated genes were identified. The number of hyper-methylated DMGs was almost 1.33–1.58 times the number of hypo-methylated DMGs in grass carp fed with a high-carbohydrate diet. To understand the effect of carbohydrate-induced DNA methylation variations on the metabolic characteristics and the phenotypes in grass carp, the DNA methylation profiles of critical metabolism-related pathways, including carbohydrate metabolism, insulin pathway, lipid metabolism, adipocytokine signaling pathway and AMPK signaling pathway, were analyzed.

Insulin is regarded as the primary regulator of glucose concentration in blood. It stimulates the uptake of glucose in muscle and fat tissues and suppresses glucose production in the liver. The initial molecular signal of insulin action contains the activation of insulin receptor tyrosine kinase and then brings about phosphorylation of insulin receptor substrates on tyrosine residues (32). In grass carp, dietary carbohydrate significantly changed the DNA methylation of genes involved in insulin signaling pathway, including insulin, insulin receptors, insulin receptor subsrate-1 (*irs1*), phosphoinositide 3 kinase (*pi3k*), and their downstream factors. Furthermore, a high-carbohydrate diet remarkably increased the serum insulin concentrations and the hepatic transcript levels of *insrb* gene in grass carp. However, the increased secretion of insulin did not suppress blood glucose and lipid deposition, suggesting that the high-carbohydrate diet might cause symptoms of “insulin resistance” in grass carp. In carnivorous fish, glucose intolerance was usually considered as a consequence of poor peripheral insulin action or possibly “insulin resistance” (33). In mammals, interleukin-6 (IL6) and tumor necrosis factor have been proven to induce hepatic insulin resistance through the suppression of the cytokine signaling (SOCS3) pathway (34, 35). The subsequent studies indicated that nutrition content in a diet could result in the DNA methylation changes of these genes (36, 37). In grass carp, KEGG enrichment analysis suggested that TGF-beta signaling pathway was the most significantly correlated pathway. In addition, DNA methylation of *il6, tnf*, and *socs3* genes were impacted by a high-carbohydrate diet intake; however, the gene expression levels of *socs3* were not affected. However, more evidence should be provided to confirm the speculation that a high-carbohydrate diet could generate “insulin resistance” by epigenetic changes in fish.

In addition, the hepatic transcript levels of key glucose metabolism genes were impacted by dietary carbohydrate levels via DNA methylation regulation. In the present study, the DNA methylation profiles of key glucose enzymes, including *gs, gck, g6pc a.1, pepck, pk, phosphofructokinase* (*pfk*), and fructose-1,6-bisphosphatase 1 (*fbp1*), were regulated by dietary carbohydrate levels. Compared with the control group, the mRNA expression levels of *gs* and *pk* genes were significantly decreased. Meanwhile, the mRNA expression levels of gluconeogenesis genes (*pepck* and *g6pca.1*) were significantly increased in the HC group. As a result, the serum glucose concentrations were significantly increased. These viewpoints have also been proposed in mammalian models. Increasing folic acid levels in maternal or post-weaning diets caused differential variations in promoter DNA methylation levels and mRNA expression levels of *pepck* in rats (38). In high-fat-diet-induced obese rats, hypermethylation was correlated with a decline in the hepatic transcript levels of *gck* and *pk* genes (8). In the liver of piglets born from sows fed with betaine-supplemented diet, DNA methylation in *g6pc* promoter was decreased in accordance with the higher mRNA expression levels of *g6pc* (39). In contrast, *pepck1* was significantly hypermethylated in the promoter region, corresponding to the diminished mRNA expression levels (39).

It is noteworthy that KEGG enrichment analysis showed that, in the promoter region, DMGs were significantly enriched in fatty acid metabolism, PPAR signal pathway, fatty acid biosynthesis, and adipocytokine signaling pathway. The results demonstrated that a high-carbohydrate diet could modify the epigenetic processes and contribute to an increase in lipid deposition in grass carp liver. The assumption was also supported by studies carried out in mammals. Dietary methionine regulated the lipogenic transcription factors. The promoter of *srebp1* was hypomethylated under vitamin B12-deficient conditions (40). Research in rodents indicated that betaine reduced the hepatic TG concentration by increasing the transcript levels of *ppar*α and the hypomethylation of gene promoter (41). In grass carp, a high-carbohydrate diet caused the variation of DNA methylation profiles in lipogenic transcription factors such as *srebp1, ppar*α, and *pcg1*α and significantly increased these gene expression levels. As a result, the mRNA expression levels of *acc1* were dramatically up-regulated and the hepatic transcripts of *fasn, cpt1*, and *atgl* genes were significantly reduced. In male Sprague–Dawley rats, metabolic syndrome pathogenesis caused by fructose was connected with DNA methylation status. Fructose feeding generated the hypermethylation of *ppar*α and *cpt1a* genes in the promoter regions and decreased the mRNA expression levels in these genes (9). A high-fat diet caused hepatic lipid accumulation by programming lipid-synthesizing genes through DNA methylation in the gene body of *fasn* and *acc1* in rats (42). Supplementation of methyl donor in diets modified the DNA methylation profiles of *fasn* and reduced the fatty liver in rats fed with an obesogenic diet (43).

In mammals, adipokines—secreted primarily by adipocytes—modulate lipid and glucose metabolism and insulin sensitivity (44). In the present study, the DNA methylation patterns of adipocytokine signaling pathway in grass carp were also analyzed. Leptin acts as one of the most crucial adipocytokines in metabolism. It regulates hepatic lipogenesis by inhibiting the transcript levels of key enzymes in the fatty acid synthesis pathway (45). Leptin also plays a major role in the regulation of metabolism in fish (46). In our previous study, the hepatic mRNA expression levels of *leptin a* were significantly improved by a high-fat diet and a high-carbohydrate diet in grass carp (15, 18). In mammals, DNA methylation of leptin was associated with maternal nutritional state and diet contents. In obese rats, DNA methylation of leptin promoter was related to decreased leptin levels in the circulation (47). The epigenetic alteration of leptin might be one of the mechanisms through which maternal phenotypes can program offspring health (48). Adiponectin, identified as one of the important adipokines, is able to reduce the triglyceride levels and strengthen the insulin signaling pathway in mammals (49, 50). Two different subtype receptors of adiponectin have been reported, named as AdipoR1 and AdipoR2. AdipoR2 is exclusively expressed in liver tissue (51). Binding to its receptors, adiponectin could activate the signaling pathway conduction and play a biological effect (52). Hypermethylation in adiponectin promoter suppressed its mRNA expression levels and exacerbated the metabolic diseases in obesity (53). In the present study, although the hepatic transcript levels of *leptin* were remarkably increased by a high-carbohydrate diet, there was no significant difference in DNA methylation in the promoter or the gene body regions of *leptin* or *adiponectin* between the two groups in grass carp. On the contrary, the DNA methylation levels of *leptin receptor* and *adiponectin receptor* were altered by a high-carbohydrate diet. The results indicated differences in the epigenetic regulation mechanism between mammals and fish. However, more studies in fish species should be conducted to identify the epigenetic regulation mechanisms and the differences among species.

Up to now, most knowledge of epigenetic regulation in animals has come from investigations in mammals, and researchers should be cautious when generalizing results from mammals to fish. Comparative methylome analysis across taxa exposed wide discrepancies in methylation profiles and indicated that DNA methylation might perform different functions among species (30, 54, 55). Comparative DNA methylation profiles of mouse and zebrafish likewise uncovered a high divergence between species. The global DNA methylation levels in zebrafish were significantly higher than those in rats (56). A comparative genomics analysis of carbohydrate metabolic genes between fish and human revealed that most metabolic genes were conserved in vertebrates (57). Whether the DNA methylation regulation of a single gene in different species is conserved remains an important question. Therefore, we compared the DNA methylation alterations related to glucose metabolism and obesity pathways in grass carp with the mammalian models of obesity and T2D or in a different nutritional state (Table 3). The results showed that most of the DMGs in grass carp were also regulated by DNA methylation in mammals when the nutrient states changed. The findings would lead us to reveal novel differentially methylated regions and candidate genes for glucose metabolism by breaking species boundaries.

**Table 3:** Comparison of DNA methylation alterations of metabolism-related genes with mammal models of obesity and type 2 diabetes.

**Target gene**	**Description**	**Region**		**Sample type**	**Major finding**	**References**
		**5′ flanking**	**Gene body**			
FABP	Liver type fatty acid binding protein	√	√	Human adipose	Polyunsaturated and saturated fat overfeeding induce distinct epigenetic changes, including fatty acid binding protein	(58)
LPL	Lipoprotein lipase	√	/	Rat adipose	Maternal prenatal folic acid supplementation programs offspring lipid metabolism by aberrant DNA methylation in LPL gene	(59)
ATGL	Adipose triglyceride lipase	√	/	Rat liver	Maternal prenatal folic acid supplementation programs offspring lipid metabolism by aberrant DNA methylation in ATGL gene	(59)
FASN	Fatty acid synthase	√	√	Rat liver	High-fat diet induced lipid-synthesizing genes via gene body methylation of FAS and ACC1	(42)
ACC1	Acetyl-CoA carboxylase 1	/	√	Rat liver	High-fat diet induced lipid-synthesizing genes via gene body methylation of FAS and ACC1	(42)
CPT1	Carnitine palmitoyltransferase 1Ab	√	√	Rat liver	High fructose consumption induced DNA methylation in PPARα and CPT1A promoter	(9)
FATB	Solute carrier family 27, member 1/4	/	√	–	–	–
HSL	Hormone sensitive lipase	/	√	Adipose tissue of metabolic syndrome patients	DNA methylation of LPL was associated with triglyceride concentrations in the metabolic syndrome	(60)
CS	Citrate synthase	√	√	–	–	–
GS	Glycogen synthase	√	/	–	–	–
GK	Glucokinase	√	/	Rat liver	Obese rats fed with high-fat diet revealed hypermethylation in promoter regions of hepatic gk and pk	(8)
PK	Pyruvate kinase	√	√	Rat liver	Obese rats fed with high-fat diet revealed hypermethylation in promoter regions of hepatic gk and pk	(8)
G6PC	Glucose 6-phosphatase alpha	√	/	Piglet liver	Betaine supplementation in maternal diet reduced the DNA methylation in G6PC and improved the G6PC mRNA expression	(39)
PEPCK1	Phosphoenolpyruvate carboxykinase 1	√	/	Piglet liver	Betaine supplementation in maternal diet improved the DNA methylation of PEPCK1 promoter and diminished the PEPCK1 mRNA expression	(39)
PEPCK2	Phosphoenolpyruvate carboxykinase 2	√	√	–	–	–
PFK1	6-Phosphofructokinase 1	/	√	–	–	–
PFK2	6-Phosphofructokinase 2	√	√	–	–	–
FBP1	Fructose-1,6-bisphosphatase 1	/	√	Human hepatocellular carcinoma and colon cancer	Hypermethylation of FBP1 promoter down-regulated FBP1 expression	(61)
INS	Insulin	√	/	Mouse embryonic stem cell	Ins2 was fully methylated and became demethylated as the cells differentiate into insulin-expressing cells *in vitro*	(62)
IRA	Insulin receptor A	/	√	Hypothalamus of rat	Insulin receptor promoter is vulnerable to hypermethylation due to overnutrition, probably especially glucose dependent in a dose–response manner	(63)
IRB	Insulin receptor B	/	√	–	–	–
IRS	Insulin receptor substrate	/	√	Human blood	There was no association between IRS-1 promoter methylation and type 2 diabetes between genders	(64)
SREBP1	Sterol regulatory element binding transcription factor 1	/	√	Human adipose	The promoter of SREBF1 was hypomethylated under vitamin B12-deficient conditions	(40)
IL-6	Interleukin 6	/	√	Peripheral blood mononuclear cells	The methylation status of a single CpG site in the IL6 promoter is related to IL6 mRNA expression and rheumatoid arthritis	(65)
TNFα	Tumor necrosis factor α	/	√	Human adipose	TNF α promoter methylation levels could be used as epigenetic biomarkers concerning response to low-calorie diet	(43)
LepR	Leptin receptor	/	√	–	–	–
AdipoR	Adiponectin receptor	√	/	–	–	–
SOCS3	Suppressor of cytokine signaling 3	√	/	Peripheral blood mononuclear cells	DNA methylation of SOCS3 was inversely related with metabolic syndrome	(37)
PPARα	Peroxisome proliferator-activated receptors α a	√	√	Rat liver	High fructose consumption induces DNA methylation at PPARα and CPT1A promoter	(9)
AMPK	5′-AMP-activated protein kinase	√	√	Human blood and mouse muscle	DNA methylation of AMPK gene increased after moderate-endurance exercise in humans and mice	(66)
PGC-1α	Peroxisome proliferator-activated receptor gamma coactivator 1 α	√	√	Human THP-1 monocytes and rat adipose	Supplementation with saturated fatty acids and elaidic acid induced the hypermethylation of PPARG1	(25)

## Conclusions

In conclusion, to address the knowledge gap of nutrition-related, especially glucose metabolism- and obesity-related, epigenetics in fish, our study provided an in-depth analysis of genome-wide methylation in grass carp liver. It is the first research that systematically performs a comparative analysis on the genome-wide methylation profiles in fish with different nutritional states. Although the mRNA expression levels of genes involved in glucose metabolism were significantly changed in grass carp fed with diets of different carbohydrate contents, there was no significant difference in the global genomic DNA methylation levels. Despite that, differentially methylated genes related to the regulation of metabolism pathways were found between the two groups. Furthermore, a comparison of DNA methylation alterations with mammals demonstrated that most of the DMGs in grass carp were also regulated by DNA methylation in mammals when the nutrient states changed. Our study may resolve some complexities of glucose metabolic heterogeneity among vertebrates and provide a reference for the aquaculture industry and the treatment of diabetes.

## Data Availability Statement

Publicly available datasets were analyzed in this study. The data sets used are available in the Sequence ReadArchive database (SRA accession: PRJNA558443).

## Ethics Statement

The animal study was reviewed and approved by Institutional Animal Care and Use Ethics Committee of Huazhong Agricultural University.

## Author Contributions

W-JC conceived the study, participated in the design of the study, carried out the laboratory work, participated in data analysis, and wrote the first draft of the manuscript. X-FL designed the study and contributed to the final version. X-CY and A-XL collected the source animals and assisted with establishing the experimental treatments. SH participated in the design of the study. All authors contributed to the article and approved the submitted version.

## Conflict of Interest

The authors declare that the research was conducted in the absence of any commercial or financial relationships that could be construed as a potential conflict of interest.

## Supplementary Material

Figure S1 Feature of hepatic genome-wide methylation in grass carp fed with moderate carbohydrate diet (control) or excessive carbohydrate diet (HC). **(A)** Genome-wide methylated cytosines levels. **(B)** Proportion of methylated cytosines in mCG, mCHG, and mCHH patterns. The red part represents mCG, blue represents mCHG and green represents mCHH. The sum of three parts is 100% which consists of the whole pie chart. **(C)** Canonical DNA methylation profiles of the entire transcriptional units. The canonical gene structure is defined by seven different features, denoted by the *x*-axis. a: upstream, b: first exon, c: first intron, d: internal exon, e: internal intron, f: last exon, and g: downstream. The length of each feature was normalized and divided into equal numbers of bins. Each dot denotes the mean methylation level per bin and the respective lines denote the five-bin moving average. Each feature was analyzed separately for the numbers listed in the table below the figure. The green vertical line indicates the mean location of the transcription start sites.

Figure S2 Distribution and proportion of differentially methylated genes (DMGs) that differently methylated in the promoter and the gene body regions. **(A)** Number of differentially methylated genes of mCG, mCHG, and mCHH. **(B)** Number of hyper-methylated and hypo-methylated genes.

Figure S3 Gene Ontology (GO) enrichment analysis of DMGs. **(A)** GO enrichment analysis of DMGs that differentially methylated in the promoter region. **(B)** GO enrichment analysis of DMGs that differentially methylated in the gene body region. *Y*-axis represents three domains of GO and *x*-axis represents the gene number in every pathway and processes.

Figure S4 KEGG enrichment analysis of DMGs. **(A)** Pathway analysis of DMGs differentially methylated in the promoter region. **(B)** Pathway analysis of DMGs differentially methylated in the gene body region. Y-axis represents pathways and x-axis represents the gene number in every pathway and processes.

Table S1 Data Summary and Quality Control (QC).

Table S2 Alignment statistics with reference genome.

Table S3 QC items for each sample.

## References

[1] NRC (2011). Nutrient Requirements of Fish and Shrimp. Washington, DC: National Academies Press.

[2] Shiau SY Peng CY Protein-sparing effect by carbohydrates in diets for tilapia, *Oreochromis niloticus* × O. *aureus*. Aquaculture. (1993) 117:327–34. 10.1016/0044-8486(93)90329-W

[3] Krishnan J Rohner N. Sweet fish: Fish models for the study of hyperglycemia and diabetes. J Diabetes. (2019) 11:193–203. 10.1111/1753-0407.12860 30264455

[4] Zang L Shimada Y Nishimura N. Development of a novel zebrafish model for type 2 diabetes mellitus. Sci Rep. (2017) 7:1461. 10.1038/s41598-017-01432-w 28469250 PMC5431185

[5] Kelley KM. Experimental diabetes mellitus in a teleost fish. I. Effect of complete isletectomy and subsequent hormonal treatment on metabolism in the goby, *Gillichthys mirabilis*. Endocrinology. (1993) 132:2689–95. 10.1210/endo.132.6.8504768 8504768

[6] Wright JR Abraham C Dickson BC Yang H Morrison CM. Streptozotocin dose-response curve in tilapia, a glucose-responsive teleost fish. Gen Comp Endocr. (1999) 114:431–40. 10.1006/gcen.1999.7269 10336831

[7] Riddle MR Aspiras AC Gaudenz K Peuß R Sung JY Martineau B. Insulin resistance in cavefish as an adaptation to a nutrient-limited environment. Nature. (2018) 555:647. 10.1038/nature26136 29562229 PMC5989729

[8] Jiang M Zhang Y Liu M Lan MS Fei J Fan W. Hypermethylation of hepatic glucokinase and L-type pyruvate kinase promoters in high-fat diet-induced obese rats. Endocrinology. (2011) 152:1284–9. 10.1210/en.2010-1162 21239437

[9] Ohashi K Munetsuna E Yamada H Ando Y Yamazaki M Taromaru N. High fructose consumption induces DNA methylation at PPARα and CPT1A promoter regions in the rat liver. Biochem Biophys Res Commun. (2015) 468:185–9. 10.1016/j.bbrc.2015.10.134 26519879

[10] Ling C Del Guerra S Lupi R Rönn T Granhall C Luthman H. Epigenetic regulation of PPARGC1A in human type 2 diabetic islets and effect on insulin secretion. Diabetologia. (2008) 51:615–22. 10.1007/s00125-007-0916-5 18270681 PMC2270364

[11] Yang BT Dayeh TA Volkov PA Kirkpatrick CL Malmgren S Jing X. Increased DNA methylation and decreased expression of PDX-1 in pancreatic islets from patients with type 2 diabetes. Mol Endocrinol. (2012) 26:1203–12. 10.1210/me.2012-1004 22570331 PMC5416998

[12] Yang BT Dayeh TA Kirkpatrick CL Taneera J Kumar R Groop L. Insulin promoter DNA methylation correlates negatively with insulin gene expression and positively with HbA 1c levels in human pancreatic islets. Diabetologia. (2011) 54:360–7. 10.1007/s00125-010-1967-6 21104225 PMC3017313

[13] Volkov P Bacos K Ofori JK Esguerra JLS Eliasson L Rönn T. Whole-genome bisulfite sequencing of human pancreatic islets reveals novel differentially methylated regions in type 2 diabetes pathogenesis. Diabetes. (2017) 66:1074–85. 10.2337/db16-0996 28052964

[14] Ma X Kang S. Functional implications of DNA methylation in adipose biology. Diabetes. (2019) 68:871–8. 10.2337/dbi18-0057 31010878 PMC6477906

[15] Li A Yuan X Liang XF Liu L Li J Li B Adaptations of lipid metabolism and food intake in response to low and high fat diets in juvenile grass carp (*Ctenopharyngodon idellus*). Aquaculture. (2016) 457:43–9. 10.1016/j.aquaculture.2016.01.014

[16] Gao W Liu YJ Tian LX Mai KS Liang GY Yang HJ Effect of dietary carbohydrate-to-lipid ratios on growth performance, body composition, nutrient utilization and hepatic enzymes activities of herbivorous grass carp (*Ctenopharyngodon idella*). Aquacult Nutr. (2010) 16:327–33. 10.1111/j.1365-2095.2009.00668.x

[17] Cai W Liang XF Yuan X Li A He Y He S. Genomic organization and expression of insulin receptors in grass carp, *Ctenopharyngodon idellus*. Comp Biochem Phys B. (2016) 194:51–7. 10.1016/j.cbpb.2015.11.013 26772721

[18] Cai W Liang XF Yuan X Liu L He S Li J Different strategies of grass carp (*Ctenopharyngodon idella*) responding to insufficient or excessive dietary carbohydrate. Aquaculture. (2018) 497:292–8. 10.1016/j.aquaculture.2018.07.042

[19] Mao X Cai T Olyarchuk JG Wei L. Automated genome annotation and pathway identification using the KEGG Orthology (KO) as a controlled vocabulary. Bioinformatics. (2005) 21:3787–93. 10.1093/bioinformatics/bti430 15817693

[20] Ma D Fan J Tian Y Jiang P Wang J Zhu H. Selection of reference genes for quantitative real-time PCR normalisation in largemouth bass *Micropterus salmoides* fed on alternative diets. J Fish Biol. (2019) 95:393–400. 10.1111/jfb.13991 31017661

[21] Livak KJ Schmittgen TD. Analysis of relative gene expression data using real-time quantitative PCR and the 2^ΔΔ*CT*^ method. Methods. (2001) 25:402–8. 10.1006/meth.2001.1262 11846609

[22] Weaver IC Cervoni N Champagne FA D'Alessio AC Sharma S Seckl JR. Epigenetic programming by maternal behavior. Nat Neurosci. (2004) 7:847. 10.1038/nn1276 15220929

[23] Samblas M Milagro FI Martínez A. DNA methylation markers in obesity, metabolic syndrome, and weight loss. Epigenetics. (2019) 14:421–44. 10.1080/15592294.2019.1595297 30915894 PMC6557553

[24] Hall E Volkov P Dayeh T Bacos K Rönn T Nitert MD. Effects of palmitate on genome-wide mRNA expression and DNA methylation patterns in human pancreatic islets. BMC Med. (2014) 12:103. 10.1186/1741-7015-12-103 24953961 PMC4065864

[25] Flores-Sierra J Arredondo-Guerrero M Cervantes-Paz B Rodríguez-Ríos D Alvarado-Caudillo Y Nielsen FC. The trans fatty acid elaidate affects the global DNA methylation profile of cultured cells and *in vivo*. Lipids Health Dis. (2016) 15:75. 10.1186/s12944-016-0243-2 27068706 PMC4828757

[26] Terova G Díaz N Rimoldi S Ceccotti C Gliozheni E Piferrer F. Effects of sodium butyrate treatment on histone modifications and the expression of genes related to epigenetic regulatory mechanisms and immune response in European sea bass (*Dicentrarchus Labrax*) fed a plant-based diet. PLoS One. (2016) 11:e0160332. 10.1371/journal.pone.0160332 27471849 PMC4966935

[27] Marandel L Lepais O Arbenoits E Véron V Dias K Zion M. Remodelling of the hepatic epigenetic landscape of glucose-intolerant rainbow trout (*Oncorhynchus mykiss*) by nutritional status and dietary carbohydrates. Sci Rep. (2016) 6:32187. 10.1038/srep32187 27561320 PMC4999891

[28] Akemann C Meyer DN Gurdziel K Baker TR. Developmental dioxin exposure alters the methylome of adult male zebrafish gonads. Front Genet. (2018) 9:719. 10.3389/fgene.2018.00719 30687390 PMC6336703

[29] Wan ZY Xia JH Lin G Wang L Lin VC Yue GH. Genome-wide methylation analysis identified sexually dimorphic methylated regions in hybrid tilapia. Sci Rep. (2016) 6:35903. 10.1038/srep35903 27782217 PMC5080608

[30] Zemach A McDaniel IE Silva P Zilberman D. Genome-wide evolutionary analysis of eukaryotic DNA methylation. Science. (2010) 328:916. 10.1126/science.1186366 20395474

[31] Liu J Dias K Plagnes-Juan E Veron V Panserat S Marandel L. Long-term programming effect of embryonic hypoxia exposure and high-carbohydrate diet at first feeding on glucose metabolism in juvenile rainbow trout. J Exp Biol. (2017) 220:3686–94. 10.1242/jeb.161406 28798080

[32] Whitehead JP Clark SF Ursø B James DE. Signalling through the insulin receptor. Curr Opin Cell Biol. (2000) 12:222–8. 10.1016/S0955-0674(99)00079-4 10712920

[33] Polakof S Skiba-Cassy S Choubert G Panserat S. Insulin-induced hypoglycaemia is co-ordinately regulated by liver and muscle during acute and chronic insulin stimulation in rainbow trout (*Oncorhynchus mykiss*). J Exp Biol. (2010) 213:1443–52. 10.1242/jeb.037689 20400628

[34] Kim JH Kim JE Liu H-Y Cao W Chen J. Regulation of interleukin-6-induced hepatic insulin resistance by mammalian target of rapamycin through the STAT3-SOCS3 pathway. J Biol Chem. (2008) 283:708–15. 10.1074/jbc.M708568200 17993646

[35] Kern PA Ranganathan S Li C Wood L Ranganathan G. Adipose tissue tumor necrosis factor and interleukin-6 expression in human obesity and insulin resistance. Am J Physiol Endocrinol Metab. (2001) 280:E745–E51. 10.1152/ajpendo.2001.280.5.E745 11287357

[36] Cordero P Campion J Milagro FI Goyenechea E Steemburgo T Javierre BM. Leptin and TNF-alpha promoter methylation levels measured by MSP could predict the response to a low-calorie diet. J Physiol Biochem. (2011) 67:463–70. 10.1007/s13105-011-0084-4 21465273

[37] Ali O Cerjak D Kent JW Jr James R Blangero J Carless MA. Methylation of SOCS3 is inversely associated with metabolic syndrome in an epigenome-wide association study of obesity. Epigenetics. (2016) 11:699–707. 10.1080/15592294.2016.1216284 27564309 PMC5048720

[38] Hoile SP Lillycrop KA Grenfell LR Hanson MA Burdge GC. Increasing the folic acid content of maternal or post-weaning diets induces differential changes in phosphoenolpyruvate carboxykinase mRNA expression and promoter methylation in rats. Br J Nutr. (2012) 108:852–7. 10.1017/S0007114511006155 22136740

[39] Cai D Jia Y Song H Sui S Lu J Jiang Z. Betaine supplementation in maternal diet modulates the epigenetic regulation of hepatic gluconeogenic genes in neonatal piglets. PLoS One. (2014) 9:e105504. 10.1371/journal.pone.0105504 25153319 PMC4143294

[40] Adaikalakoteswari A Finer S Voyias PD McCarthy CM Vatish M Moore J. Vitamin B 12 insufficiency induces cholesterol biosynthesis by limiting s-adenosylmethionine and modulating the methylation of SREBF1 and LDLR genes. Clin Epigenet. (2015) 7:14. 10.1186/s13148-015-0046-8 25763114 PMC4356060

[41] Wang L Chen L Tan Y Wei J Chang Y Jin T. Betaine supplement alleviates hepatic triglyceride accumulation of apolipoprotein E deficient mice via reducing methylation of peroxisomal proliferator-activated receptor alpha promoter. Lipids Health Dis. (2013) 12:34. 10.1186/1476-511X-12-34 23497035 PMC3621752

[42] Jung PM High Fat Diet Causes Hepatic Lipid Accumulation by Programming Lipid Synthesizing Genes via Gene Body Methylation. (2017). Available online at: http://hdl.handle.net/2142/99124

[43] Cordero P Gómez-Úriz AM Campion J Milagro F Martinez JA. Dietary supplementation with methyl donors reduces fatty liver and modifies the fatty acid synthase DNA methylation profile in rats fed an obesogenic diet. Genes Nutr. (2013) 8:105–13. 10.1007/s12263-012-0300-z 22648174 PMC3534997

[44] Rabe K Lehrke M Parhofer KG Broedl UC. Adipokines and insulin resistance. Mol Med. (2008) 14:741–51. 10.2119/2008-00058.Rabe 19009016 PMC2582855

[45] Cohen P Miyazaki M Socci ND Hagge-Greenberg A Liedtke W Soukas AA. Role for stearoyl-CoA desaturase-1 in leptin-mediated weight loss. Science. (2002) 297:240–3. 10.1126/science.1071527 12114623

[46] Dalman MR Liu Q King MD Bagatto B Londraville RL. Leptin expression affects metabolic rate in zebrafish embryos (*D. rerio*). Front Physiol. (2013) 4:160. 10.3389/fphys.2013.00160 23847542 PMC3696835

[47] Milagro F Campion J Garcia-Diaz D Goyenechea E Paternain L Martinez J. High fat diet-induced obesity modifies the methylation pattern of leptin promoter in rats. J Physiol Biochem. (2009) 65:1–9. 10.1007/BF03165964 19588726

[48] Lesseur C Armstrong DA Paquette AG Li Z Padbury JF Marsit C. Maternal obesity and gestational diabetes are associated with placental leptin DNA methylation. Am J Obstet Gynecol. (2014) 211:651.e1–654.e9. 10.1016/j.ajog.2014.06.037 24954653 PMC4254188

[49] Pajvani UB Scherer PE. Adiponectin: systemic contributor to insulin sensitivity. Curr Diabetes Rep. (2003) 3:207–13. 10.1007/s11892-003-0065-2 12762967

[50] Goldstein BJ Scalia R. Adiponectin: a novel adipokine linking adipocytes and vascular function. J Clin Endocr Metab. (2004) 89:2563–8. 10.1210/jc.2004-0518 15181024

[51] Kadowaki T Yamauchi T Adiponectin adiponectin receptors. Endocr Rev. (2005) 26:439–51. 10.1210/er.2005-0005 15897298

[52] Yoon MJ Lee GY Chung J-J Ahn YH Hong SH Kim JB. Adiponectin increases fatty acid oxidation in skeletal muscle cells by sequential activation of AMP-activated protein kinase, p38 mitogen-activated protein kinase, and peroxisome proliferator-activated receptor α. Diabetes. (2006) 55:2562–70. 10.2337/db05-1322 16936205

[53] Kim AY Park YJ Pan X Shin KC Kwak S-H Bassas AF. Obesity-induced DNA hypermethylation of the adiponectin gene mediates insulin resistance. Nat Commun. (2015) 6:7585. 10.1038/ncomms8585 26139044 PMC4506505

[54] Lee TF Zhai J Meyers BC. Conservation and divergence in eukaryotic DNA methylation. Proc Natl Acad Sci U S A. (2010) 107:9027–8. 10.1073/pnas.1005440107 20457928 PMC2889049

[55] Feng S Cokus SJ Zhang X Chen P-Y Bostick M Goll MG. Conservation and divergence of methylation patterning in plants and animals. Proc Natl Acad Sci U S A. (2010) 107:8689–94. 10.1073/pnas.1002720107 20395551 PMC2889301

[56] Zhang C Hoshida Y Sadler K. Comparative epigenomic profiling of the DNA methylome in mouse and zebrafish uncovers high interspecies divergence. Front Genet. (2016) 7:110. 10.3389/fgene.2016.00110 27379160 PMC4911366

[57] Zhang Y Qin C Yang L Lu R Zhao X Nie G. A comparative genomics study of carbohydrate/glucose metabolic genes: from fish to mammals. BMC Genomics. (2018) 19:246. 10.1186/s12864-018-4647-4 29642853 PMC5896114

[58] Perfilyev A Dahlman I Gillberg L Rosqvist F Iggman D Volkov P. Impact of polyunsaturated and saturated fat overfeeding on the DNA-methylation pattern in human adipose tissue: a randomized controlled trial. Am J Clin Nutr. (2017) 105:991–1000. 10.3945/ajcn.116.143164 28275132

[59] Yang X Huang Y Sun C Li J. Maternal prenatal folic acid supplementation programs offspring lipid metabolism by aberrant DNA methylation in hepatic ATGL and adipose LPL in rats. Nutrients. (2017) 9:935. 10.3390/nu9090935 28846595 PMC5622695

[60] Castellano-Castillo D Moreno-Indias I Fernández-García JC Alcaide-Torres J Moreno-Santos I Ocaña L. Adipose tissue LPL methylation is associated with triglyceride concentrations in the metabolic syndrome. Clin Chem. (2018) 64:210–8. 10.1373/clinchem.2017.277921 29046332

[61] Chen M Zhang J Li N Qian Z Zhu M Li Q. Promoter hypermethylation mediated downregulation of FBP1 in human hepatocellular carcinoma and colon cancer. PLoS One. (2011) 6:e25564. 10.1371/journal.pone.0025564 22039417 PMC3198434

[62] Kuroda A Rauch TA Todorov I Ku HT Al-Abdullah IH Kandeel F. Insulin gene expression is regulated by DNA methylation. PloS One. (2009) 4:e6953. 10.1371/journal.pone.0006953 19742322 PMC2735004

[63] Plagemann A Roepke K Harder T Brunn M Harder A Wittrock-Staar M. Epigenetic malprogramming of the insulin receptor promoter due to developmental overfeeding. J Perinat Med. (2010) 38:393–400. 10.1515/jpm.2010.051 20443665

[64] Ma J Cheng J Wang L Wang H Xu L Liu P. No association between IRS-1 promoter methylation and type 2 diabetes. Mol Med Rep. (2013) 8:949–53. 10.3892/mmr.2013.1569 23828647

[65] Nile CJ Read RC Akil M Duff GW Wilson AG. Methylation status of a single CpG site in the IL6 promoter is related to IL6 messenger RNA levels and rheumatoid arthritis. Arthritis Rheum. (2008) 58:2686–93. 10.1002/art.23758 18759290

[66] King-Himmelreich TS Schramm S Wolters MC Schmetzer J Möser CV Knothe C. The impact of endurance exercise on global and AMPK gene-specific DNA methylation. Biochem Biophys Res Commun. (2016) 474:284–90. 10.1016/j.bbrc.2016.04.078 27103439

