# Supplementary figures and images for: Changes of DNA Methylation Pattern in Metabolic Pathways Induced by High-Carbohydrate Diet Contribute to Hyperglycemia and Fat Deposition in Grass Carp (Ctenopharyngodon idellus)
Source: Front Endocrinol (Lausanne). 2020 Jul 10;11:398. doi: 10.3389/fendo.2020.00398 (PMC7381294; doi:10.3389/fendo.2020.00398)

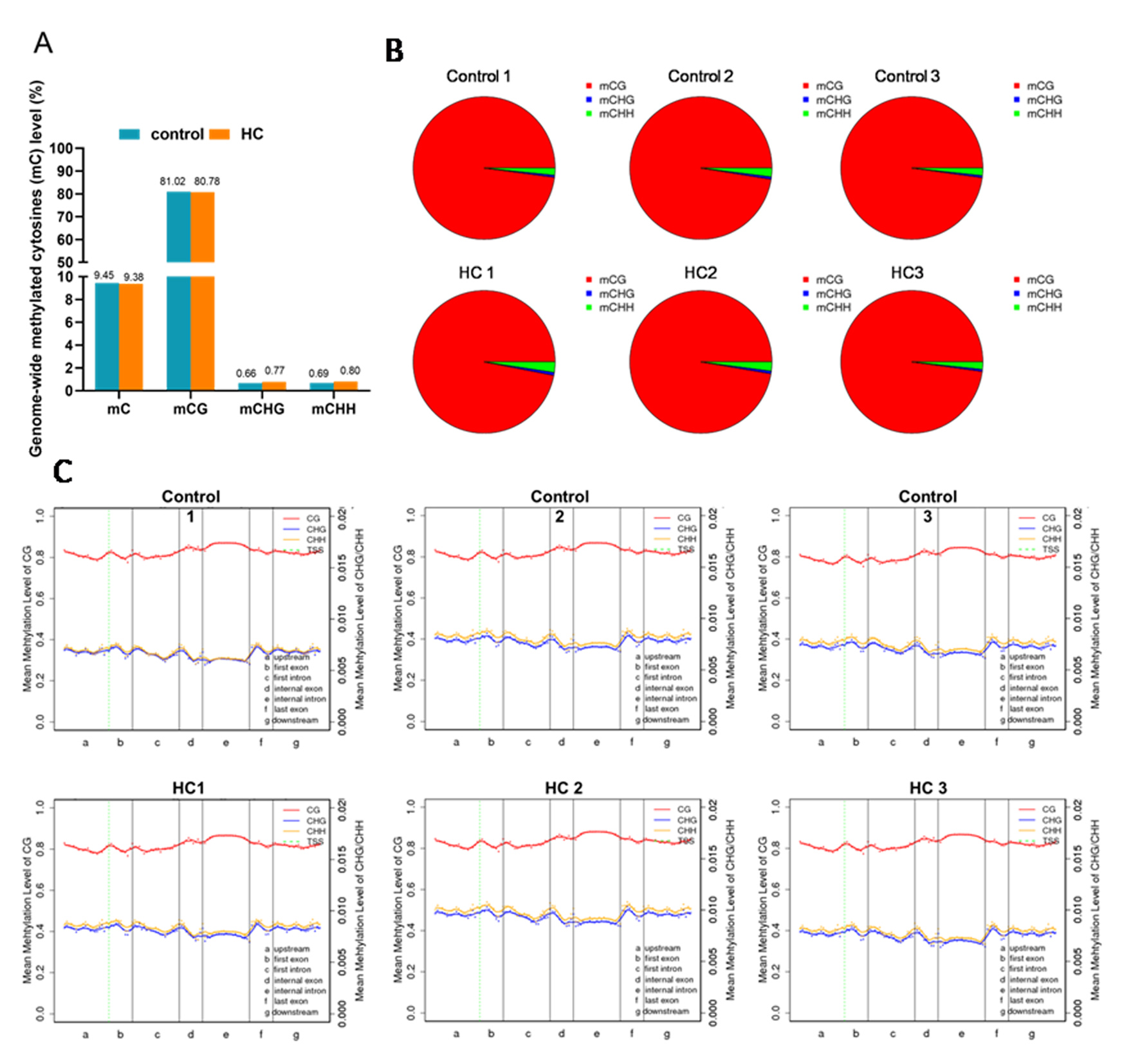

Supplement: Figure S1 — Feature of hepatic genome-wide methylation in grass carp fed with moderate carbohydrate diet (control) or excessive carbohydrate diet (HC). (A) Genome-wide methylated cytosines levels. (B) Proportion of methylated cytosines in mCG, mCHG, and mCHH patterns. The red part represents mCG, blue represents mCHG and green represents mCHH. The sum of three parts is 100% which consists of the whole pie chart. (C) Canonical DNA methylation profiles of the entire transcriptional units. The canonical gene structure is defined by seven different features, denoted by the x-axis. a: upstream, b: first exon, c: first intron, d: internal exon, e: internal intron, f: last exon, and g: downstream. The length of each feature was normalized and divided into equal numbers of bins. Each dot denotes the mean methylation level per bin and the respective lines denote the five-bin moving average. Each feature was analyzed separately for the numbers listed in the table below the figure. The green vertical line indicates the mean location of the transcription start sites. [file Image_1.JPEG]

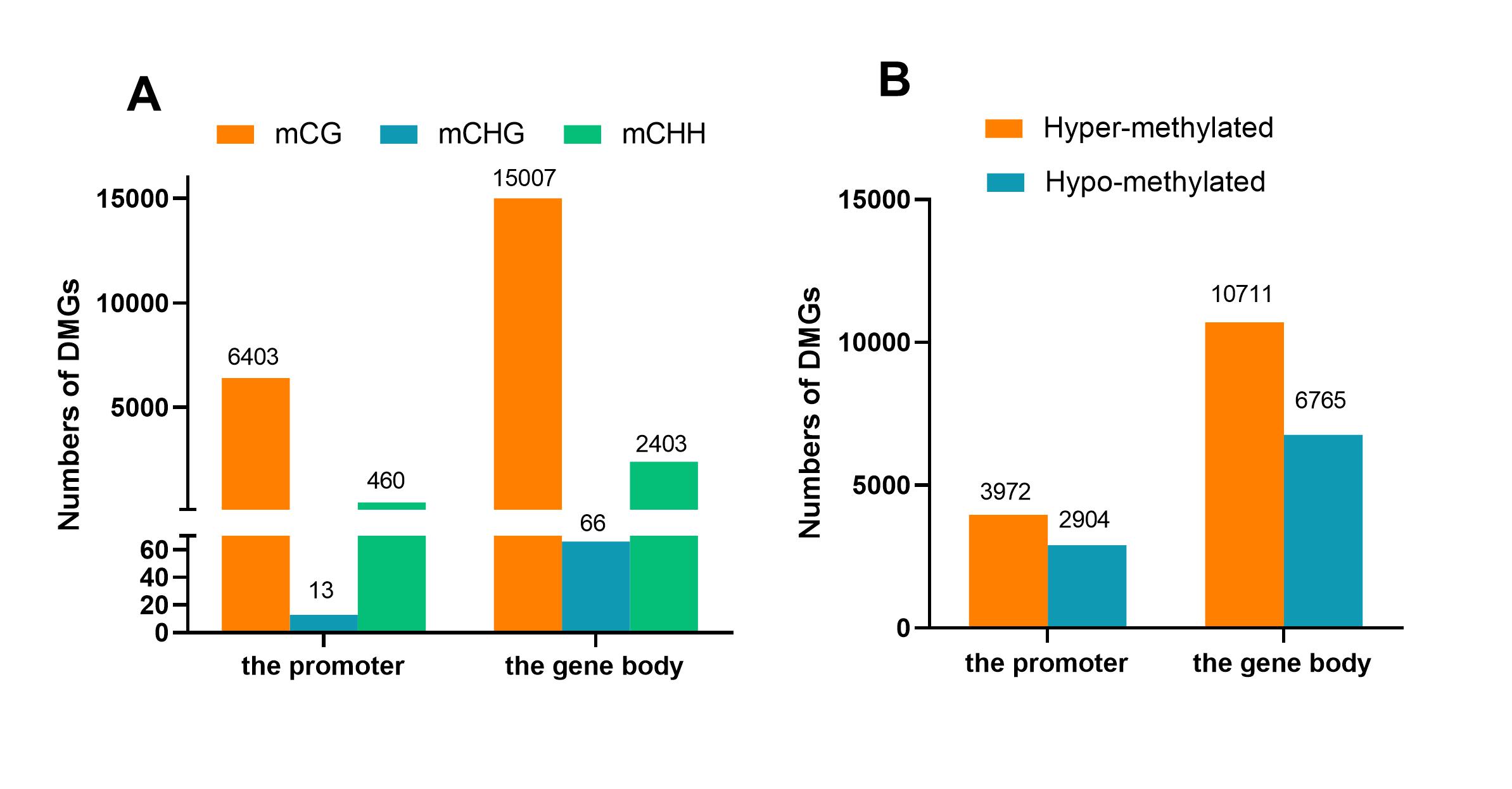

Supplement: Figure S2 — Distribution and proportion of differentially methylated genes (DMGs) that differently methylated in the promoter and the gene body regions. (A) Number of differentially methylated genes of mCG, mCHG, and mCHH. (B) Number of hyper-methylated and hypo-methylated genes. [file Image_2.JPEG]

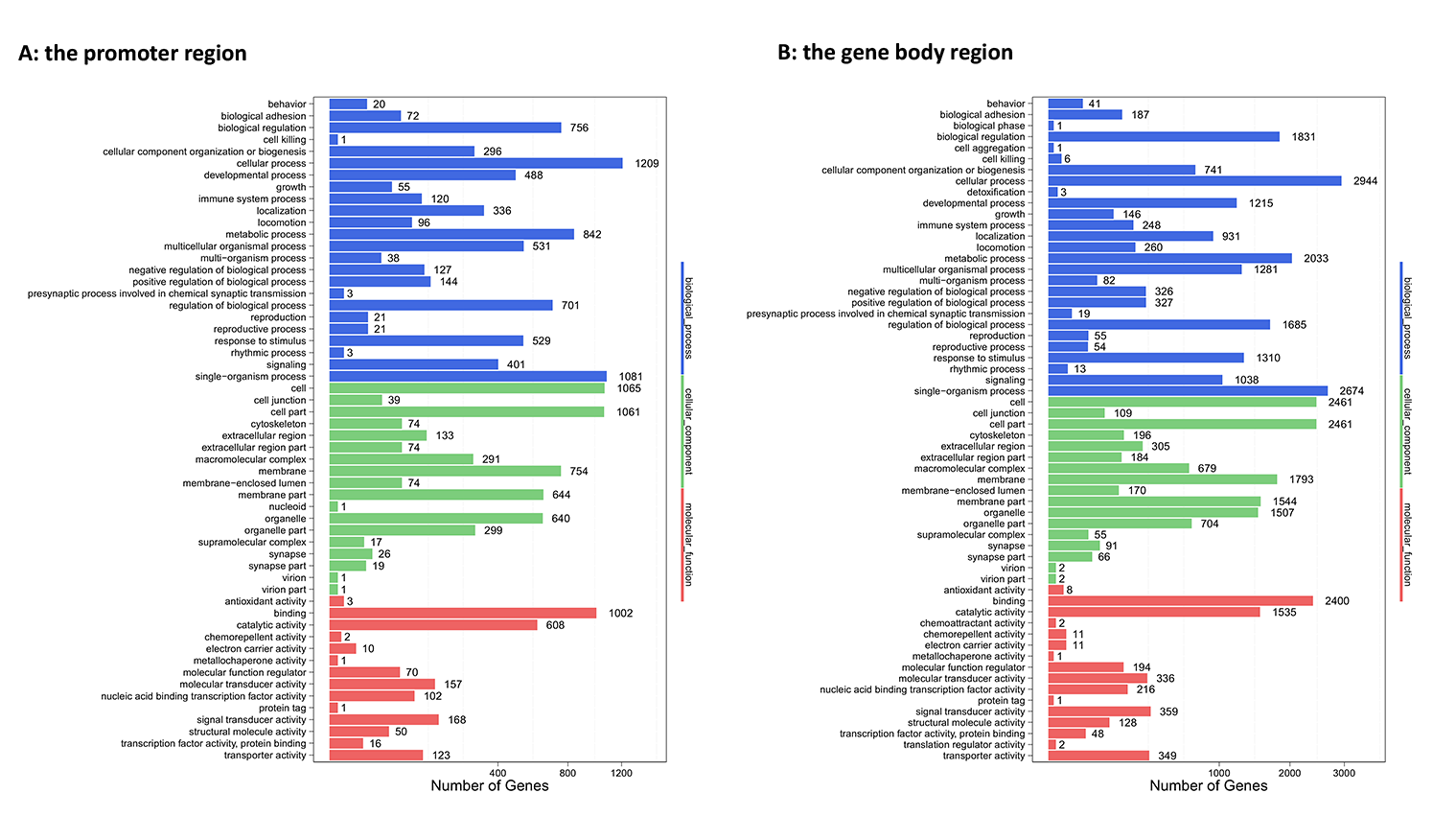

Supplement: Figure S3 — Gene Ontology (GO) enrichment analysis of DMGs. (A) GO enrichment analysis of DMGs that differentially methylated in the promoter region. (B) GO enrichment analysis of DMGs that differentially methylated in the gene body region. Y-axis represents three domains of GO and x-axis represents the gene number in every pathway and processes. [file Image_3.TIF]

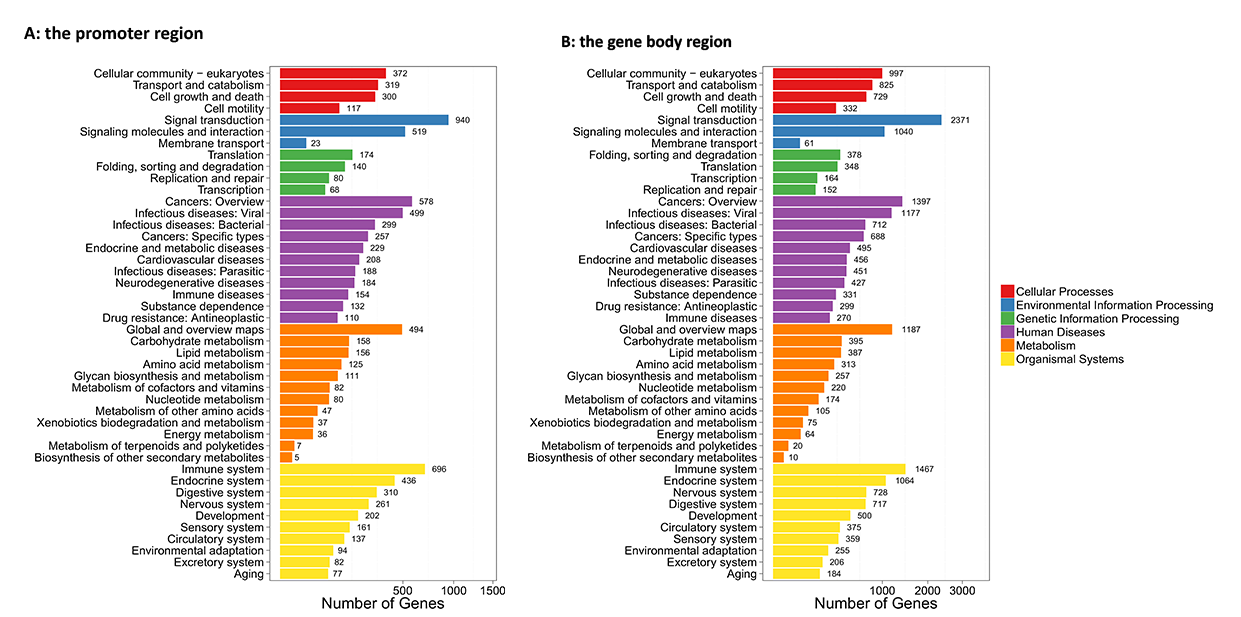

Supplement: Figure S4 — KEGG enrichment analysis of DMGs. (A) Pathway analysis of DMGs differentially methylated in the promoter region. (B) Pathway analysis of DMGs differentially methylated in the gene body region. Y-axis represents pathways and x-axis represents the gene number in every pathway and processes. [file Image_4.TIF]
