# Supplementary material for: Changes of DNA Methylation Pattern in Metabolic Pathways Induced by High-Carbohydrate Diet Contribute to Hyperglycemia and Fat Deposition in Grass Carp (Ctenopharyngodon idellus)
Source: Front Endocrinol (Lausanne). 2020 Jul 10;11:398. doi: 10.3389/fendo.2020.00398 (PMC7381294; doi:10.3389/fendo.2020.00398)
Supplement: Table S1 — Data Summary and Quality Control (QC). [file Data_Sheet_1.docx]

Table S1 Data Summary and Quality Control (QC)

| Sample ID | Fragment Length(bp) | Sequencing Strategy | Clean Reads Number | Clean Data Size(bp) | Clean Rate(%) |
| --- | --- | --- | --- | --- | --- |
| control1 | 100~500bp | PE150 | 277538024 | 41630703600 | 92.93 |
| control2 | 100~500bp | PE150 | 221313940 | 33197091000 | 94.15 |
| control3 | 100~500bp | PE150 | 225305512 | 33795826800 | 92.39 |
| HC1 | 100~500bp | PE150 | 300636196 | 45095429400 | 94.72 |
| HC2 | 100~500bp | PE150 | 212057894 | 31808684100 | 94.27 |
| HC3 | 100~500bp | PE150 | 221943860 | 33291579000 | 92.69 |

Clean Rate (%)= Clean Data Size (bp)/Raw Data Size (bp)

Table S2 Alignment statistics with reference genome

| Sample ID | Mapped Reads | Mapping Rate (%) | Uniquely Mapped Reads | Uniquely Mapping Rate (%) | Bisulfite Conversion Rate (%) |
| --- | --- | --- | --- | --- | --- |
| control1 | 277538024 | 86.70 | 229454257 | 82.67 | 99.61 |
| control2 | 221313940 | 84.84 | 179476208 | 81.10 | 99.51 |
| control3 | 225305512 | 83.52 | 180026021 | 79.90 | 99.56 |
| HC1 | 300636196 | 79.62 | 229740514 | 76.42 | 99.46 |
| HC2 | 212057894 | 87.31 | 176784462 | 83.37 | 99.35 |
| HC3 | 221943860 | 85.39 | 181029236 | 81.57 | 99.52 |

Table S3 QC items for each sample

| Sample ID | Clean Reads Q20 Rate (%) | Mapping Rate (%) | Bisulfite Conversion Rate (%) | Duplication Rate (%) | Average Depth (X) | Coverage (%) |
| --- | --- | --- | --- | --- | --- | --- |
| control1 | 98.56;98.14(Pass) | 86.70(Pass) | 99.61(Pass) | 8.78(Pass) | 33.53 | 90.009 |
| control2 | 98.79;98.87(Pass) | 84.84(Pass) | 99.51(Pass) | 12.67(Pass) | 25.08 | 89.973 |
| control3 | 98.58;98.13(Pass) | 83.52(Pass) | 99.56(Pass) | 7.88(Pass) | 26.62 | 89.958 |
| HC1 | 98.76;98.96(Pass) | 79.62(Pass) | 99.46(Pass) | 16.24(Pass) | 30.64 | 90.593 |
| HC2 | 98.74;98.90(Pass) | 87.31(Pass) | 99.35(Pass) | 11.86(Pass) | 24.94 | 89.734 |
| HC3 | 98.52;98.18(Pass) | 85.39(Pass) | 99.52(Pass) | 7.40(Pass) | 26.89 | 89.846 |
